# A novel polymorphic repeat in the upstream regulatory region of the estrogen-induced gene *EIG121* is not associated with the risk of developing breast or endometrial cancer

**DOI:** 10.1186/s13104-016-2086-3

**Published:** 2016-05-26

**Authors:** Katherine A. Bolton, Elizabeth G. Holliday, John Attia, Nikola A. Bowden, Kelly A. Avery-Kiejda, Rodney J. Scott

**Affiliations:** Centre for Bioinformatics, Biomarker Discovery and Information-Based Medicine, Hunter Medical Research Institute, Newcastle, NSW Australia; Priority Research Centre for Cancer, School of Biomedical Sciences and Pharmacy, Faculty of Health and Medicine, University of Newcastle, Newcastle, NSW Australia; Centre for Clinical Epidemiology and Biostatistics, School of Medicine and Public Health, Faculty of Health and Medicine, University of Newcastle, Newcastle, NSW Australia; Clinical Research Design, IT and Statistical Support Unit, Hunter Medical Research Institute, Newcastle, NSW Australia; Molecular Medicine, Pathology North, John Hunter Hospital, Newcastle, NSW Australia; Head of the Discipline of Medical Genetics, School of Biomedical Sciences and Pharmacy, Faculty of Health and Medicine, University of Newcastle, University Drive, Callaghan, NSW 2308 Australia

**Keywords:** *EIG121*, *KIAA1324*, Short tandem repeats, STR, Microsatellites, Regulatory region, Breast cancer, Endometrial cancer, Cancer risk

## Abstract

**Background:**

The estrogen-induced gene 121 (*EIG121*) has been associated with breast and endometrial cancers, but its mechanism of action remains unknown. In a genome-wide search for tandem repeats, we found that *EIG121* contains a short tandem repeat (STR) in its upstream regulatory region which has the potential to alter gene expression. The presence of this STR has not previously been analysed in relation to breast or endometrial cancer risk.

**Results:**

In this study, the lengths of this STR were determined by PCR, fragment analysis and sequencing using DNA from 223 breast cancer patients, 204 endometrial cancer patients and 220 healthy controls to determine if they were associated with the risk of developing breast or endometrial cancer. We found this repeat to be highly variable with the number of copies of the AG motif ranging from 27 to 72 and having a bimodal distribution. No statistically significant association was identified between the length of this STR and the risk of developing breast or endometrial cancer or age at diagnosis.

**Conclusions:**

The STR in the upstream regulatory region of *EIG121* is highly polymorphic, but is not associated with the risk of developing breast or endometrial cancer in the cohorts analysed here. While this polymorphic STR in the regulatory region of *EIG121* appears to have no impact on the risk of developing breast or endometrial cancer, its association with disease recurrence or overall survival remains to be determined.

**Electronic supplementary material:**

The online version of this article (doi:10.1186/s13104-016-2086-3) contains supplementary material, which is available to authorized users.

## Findings

The recent emphasis on high-throughput assays in the search to find genetic variants responsible for familial cancer risk has failed to account for a significant proportion of cases. Instead, the use of genome-wide association studies and next-generation sequencing has revealed variants that account for a small portion of heritability and has now resulted in the phrase “missing heritability” to explain that which remains unaccounted for [1, 2]. Another form of genetic variation that has been over-looked, largely due to their inability to be analysed on a large scale, is that of variable tandem repeats (TRs) which are common throughout the human genome and highly mutable [2]. TRs may be drivers of phenotypic variation as they are known to be the cause of several neurological disorders and are associated with complex diseases such as diabetes and cancer [3].

In a recent study we identified short tandem repeats (STRs) in the upstream regulatory region of genes that are candidates for conferring cancer risk [4]. One such STR is a dinucleotide AG repeat upstream of the estrogen-induced gene, *EIG121* (also known as *KIAA1324*). In endometrial cancer cases, *EIG121* is highly induced by estrogen in the endometrium and differentially expressed in endometrial cancer types [5, 6]. Studies suggest that EIG121, a transmembrane protein, has an important cellular function, as it is highly conserved across species and confers survival upon cells that have been starved of nutrients or exposed to cytotoxic chemotherapeutics [7]. Our analysis from publicly-available datasets, using the Oncomine™ Platform (http://www.oncomine.com), shows *EIG121* to be over-expressed in breast cancer compared to other cancer types (Additional file 1: Table S1; [8]) and compared to normal breast tissue (Additional file 2: Table S2; [9, 10]).

Breast and endometrial cancers are estrogen-driven malignancies, and in both diseases, higher expression of estrogen-induced genes is associated with tumours that tend to be low-grade and less aggressive [5, 11] suggesting involvement of these genes in cancer risk and/or development. As *EIG121* has already been associated with estrogen levels and cancer, we analysed the variability of this newly identified STR in a series of breast and endometrial cancer cases and in a healthy control population to determine if there was any association between its length and the risk of developing these estrogen-driven cancers.

This study included 223 breast cancer cases, 204 endometrial cancer cases and 220 healthy controls from whom blood-derived genomic DNA had been collected for previous studies in Newcastle, New South Wales (NSW), Australia [12–14]. Study participant demographics are shown in Table 1. All participants provided written informed consent for the samples to be used for research.

**Table 1:** Demographic characteristics of the participants used in this study

Characteristic	Breast cancer (*n* = 223)	Endometrial cancer (*n* = 204)	Healthy controls (*n* = 220)
Sex	All female	All female	All female
Age (at ascertainment; in years)			
Range	N/A	40–92	67–86
Median		68	73
Mean (SD)		67.9 (9.5)	73.4 (4.6)
Age (at diagnosis; in years)			
Range	22–57	37–86	N/A
Median	41	63.5	
Mean (SD)	39.8 (7.3)	63.2 (9.0)	
BMI (in kg/m^2^)^a^			
Range	N/A	16.9–66.6	17.4–47.1
Median		30.0	27.9
Mean (SD)		31.3 (7.8)	28.5 (5.3)
Underweight (BMI < 18.5)		*n* = 1	*n* = 1
Normal (18.5 ≤ BMI < 25)		*n* = 37	*n* = 58
Overweight (25 ≤ BMI < 30)		*n* = 56	*n* = 91
Obese (BMI ≥ 30)		*n* = 94	*n* = 70
Not specified		*n* = 16	*n* = 0

^a^ *BMI* body mass index

The STR (a dinucleotide AG repeat) situated 518 bp upstream of the transcription start site for *EIG121* was genotyped by polymerase chain reaction (PCR) and fragment analysis using forward (5′-aggctaatccaggagaatctcttg-3′) and reverse (5′-aggctaatccaggagaatctcttg-3′) primers designed to amplify a 232 bp length fragment. PCR was performed with Platinum *Taq* DNA Polymerase High Fidelity (Invitrogen), an annealing temperature of 61 °C and 1.5 mM MgSO_4_. Fragment analysis was conducted on the ABI3730 DNA Analyzer (Applied Biosystems (AB)) after denaturation in the presence of HiDi Formamide (AB) and GeneScan 600 LIZ Size Standard (AB). The resulting electropherograms were analysed using Peak Scanner v1.0 software (AB). Sanger sequencing [12] on at least 10 % of each sample cohort, using the same primer sequences as described above, confirmed STR lengths. A line of best fit was generated to correct lengths obtained from fragment analysis as described by Pasqualotto and co-workers [15].

Statistical analyses were performed using the Stata 11.1 software package (StataCorp LP, College Station, TX, USA) and involved non-parametric Mann–Whitney U tests, Cox proportional hazard regression, Pearson’s Chi squared and Fisher’s exact tests. The significance levels of all tests were set at *p* value < 0.05 (two-sided) and corrected for multiple comparisons using the Bonferroni method.

**Fig. 1:**
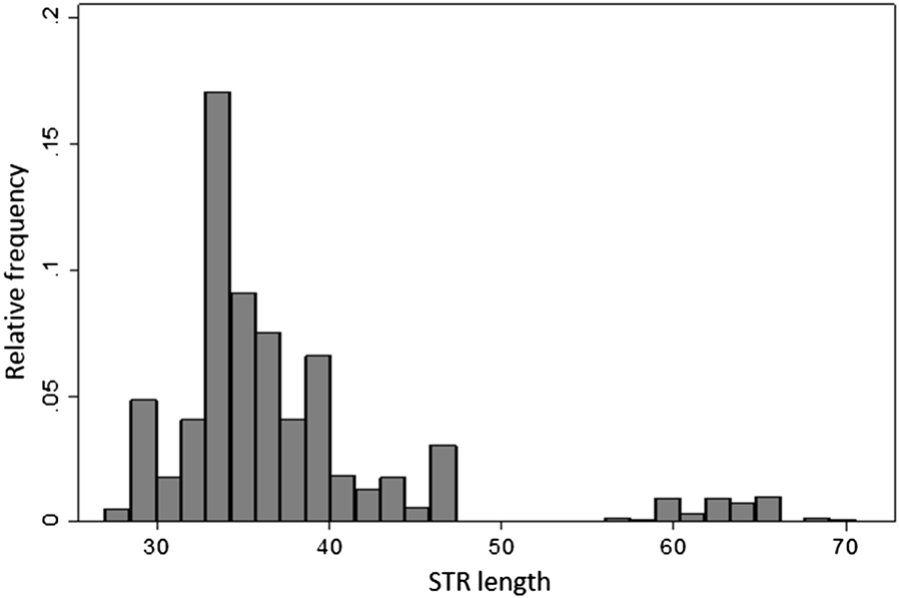
*Histogram* showing the bimodal distribution of *EIG121* STR lengths across all three cohorts (breast cancer, endometrial cancer and healthy control samples)

Based on genotyping results, the AG repeat in the upstream regulatory region of *EIG121* was highly variable, and showed a bimodal distribution of lengths with sizes ranging from 27 to 72 copies across all three cohorts (Fig. 1). The mean values for number of copies of the AG motif were 37.14, 38.34 and 37.55 for the breast cancer, endometrial cancer and healthy control cohorts respectively and the median was 35 for all three cohorts.

A non-parametric Mann–Whitney U test was used to test for any association between STR lengths, using both allele lengths for each individual, and cancer types. This demonstrated a lack of association between STR length and breast and endometrial cancers when compared to healthy controls (Table 2). To test if there was any association between STR lengths and age at diagnosis of the cancers, Cox proportional hazard regression was performed. This showed no association between STR length and age at diagnosis for breast and endometrial cancers, including when BMI was taken into account for endometrial cancer (Table 2). BMI data was not available for the breast cancer cohort. When allelic (short (S) vs long (L)) and genotypic (SS, SL and LL) analyses were performed, using a threshold of 50 copies for calling short and long alleles, there were no statistically significant associations for either cancer type (Table 2). In the breast cancer cohort, resulting *p* values for the allelic and genotypic analyses (*p* = 0.185 and *p* = 0.102, respectively; Table 2) are inclined towards a weak association between STR length and breast cancer risk. Further analysis of larger sample sizes or breast cancer subtypes would be required to confirm any possible association.

**Table 2:** Hazard ratios (HR), 95 % confidence intervals (CI) and *p* values for breast and endometrial cancer analysis in relation to *EIG121* STR lengths

Category	Statistical test	Breast cancer		Endometrial cancer	
		HR (95 % CI)	*p* value	HR (95 % CI)	*p* value
Both allele lengths with cancer risk	Mann–Whitney U test	N/A	0.985	N/A	0.262
Both allele lengths with age at diagnosis	Cox proportional hazard regression	0.994 (0.981–1.007)	0.343	1.006 (0.995–1.017)	0.304
Both allele lengths with age at diagnosis (BMI considered)	Cox proportional hazard regression	N/A	N/A	1.003 (0.992–1.015)	0.572
Allelic analysis (S/L)	Pearson’s Chi squared test	N/A	0.185	N/A	0.393
Genotypic analysis (SS/SL/LL)	Fisher’s exact test	N/A	0.102	N/A	0.545

In conclusion, the AG dinucleotide repeat in the upstream regulatory region of *EIG121* is a highly polymorphic STR, making it a variable genetic element with the potential to influence the expression of *EIG121* and subsequently impact disease risk and/or severity. No statistically significant association was identified between the length of this dinucleotide repeat and the age at diagnosis or risk of developing breast or endometrial cancer in the cohorts analysed. Hence, while this STR in the regulatory region of *EIG121* is highly polymorphic, it is unlikely to be associated with the risk of developing breast or endometrial cancer. We cannot exclude its involvement in recurrence or overall survival as this information was not available for this study.

## Additional files

10.1186/s13104-016-2086-3Statistics from analysis of the expression of *EIG121* (*KIAA1324*) from Oncomine™ Platform comparisons in multi-cancer datasets (http://www.oncomine.com). The values shown refer to over-expression of *EIG121* in breast cancer cases relative to other cancer types. The total number of samples, *n,* in each dataset are shown.
10.1186/s13104-016-2086-3Statistics from analysis of the expression of *EIG121* (*KIAA1324*) from Oncomine™ Platform comparisons of breast cancer versus normal breast samples (http://www.oncomine.com). The values shown refer to over-expression of *EIG121* in breast cancer cases compared to normal breast. The number (*n*) of breast cancer cases and normal breast samples are shown for each dataset.
