# Supplementary material for: A novel polymorphic repeat in the upstream regulatory region of the estrogen-induced gene EIG121 is not associated with the risk of developing breast or endometrial cancer
Source: BMC Res Notes. 2016 May 26;9:287. doi: 10.1186/s13104-016-2086-3 (PMC4882813; doi:10.1186/s13104-016-2086-3)
Supplement: Supplementary file 1 — 10.1186/s13104-016-2086-3Statistics from analysis of the expression of EIG121 (KIAA1324) from Oncomine™ Platform comparisons in multi-cancer datasets (http://www.oncomine.com). The values shown refer to over-expression of EIG121 in breast cancer cases relative to other cancer types. The total number of samples, n, in each dataset are shown. [file 13104_2016_2086_MOESM1_ESM.docx]

**Table S1**. **Statistics from analysis of the expression of *EIG121* (*KIAA1324*) from Oncomine™ Platform comparisons in multi-cancer datasets (www.oncomine.com).** The values shown refer to over-expression of *EIG121* in breast cancer cases relative to other cancer types. The total number of samples, *n,* in each dataset are shown.

| **Cancer Dataset** | **Cancer Type** | ***n* (Samples)** | ***p*-value (Cancer vs Cancer)** | ***t*-test (Cancer vs Cancer)** | **Fold Change** | **Gene Ranking** | **Dataset Reference** |
| --- | --- | --- | --- | --- | --- | --- | --- |
| Bittner Multi-cancer | Breast vs 15 other cancer types ^a^ | 1,911 | 9.85E-78 | 21.137 | 9.250 | 34 (in top 1%) | Not Published 2006/01/01 |
| Yu Multi-cancer | Breast vs 5 other cancer types ^b^ | 341 | 3.99E-34 | 15.491 | 4.198 | 26 (in top 1%) | PLoS Genet 2008/07/18 [8] |

^a^ Analysis of the Bittner Multi-cancer dataset for *EIG121* mRNA levels were across the following cancer types: 1. Breast cancer (*n*=328) 2. Bladder cancer (*n*=32) 3. Brain and CNS cancer (*n*=4) 4. Cervical cancer (*n*=35) 5. Colorectal cancer (*n*=330) 6. Oesophageal cancer (*n*=7) 7. Gastric cancer (*n*=7) 8. Head and neck cancer (*n*=41) 9. Kidney cancer (*n*=254) 10. Liver cancer (*n*=11) 11. Lung cancer (*n*=107) 12. Lymphoma (*n*=19) 13. Ovarian cancer (*n*=166) 14. Pancreatic cancer (*n*=19) 15. Prostate cancer (*n*=59) 16. Sarcoma (*n*=49).

^b^ Analysis of the Yu Multi-cancer dataset for *EIG121* mRNA levels were across the following cancer types: 1. Breast cancer (*n*=183) 2. Colorectal cancer (*n*=9) 3. Oesophageal cancer (*n*=16) 4. Head and neck cancer (*n*=35) 5. Liver cancer (*n*=9) 6. Lung cancer (*n*=18).
