# Supplementary material for: A novel polymorphic repeat in the upstream regulatory region of the estrogen-induced gene EIG121 is not associated with the risk of developing breast or endometrial cancer
Source: BMC Res Notes. 2016 May 26;9:287. doi: 10.1186/s13104-016-2086-3 (PMC4882813; doi:10.1186/s13104-016-2086-3)
Supplement: Supplementary file 2 — 10.1186/s13104-016-2086-3Statistics from analysis of the expression of EIG121 (KIAA1324) from Oncomine™ Platform comparisons of breast cancer versus normal breast samples (http://www.oncomine.com). The values shown refer to over-expression of EIG121 in breast cancer cases compared to normal breast. The number (n) of breast cancer cases and normal breast samples are shown for each dataset. [file 13104_2016_2086_MOESM2_ESM.docx]

**Table S2**. **Statistics from analysis of the expression of *EIG121* (*KIAA1324*) from Oncomine™ Platform comparisons of breast cancer versus normal breast samples (www.oncomine.com).** The values shown refer to over-expression of *EIG121* in breast cancer cases compared to normal breast. The number (*n*) of breast cancer cases and normal breast samples are shown for each dataset.

| **Cancer Dataset** | **Cancer Subtype** | ***n* (Case / Normal)** | ***p*-value (Cancer vs Normal)** | ***t*-test (Cancer vs Normal)** | **Fold Change** | **Gene Ranking** | **Dataset Reference** |
| --- | --- | --- | --- | --- | --- | --- | --- |
| Curtis Breast | Invasive Lobular Breast Carcinoma | 148 / 144 | 2.24E-19 | 9.683 | 2.902 | 1912 (in top 10%) | Nature 2012/04/18  [9] |
| Finak Breast | Invasive Breast Carcinoma Stroma | 53 / 6 | 6.57E-18 | 15.597 | 9.068 | 512 (in top 3%) | Nat Med 2008/05/01 [10] |
| TCGA Breast | Invasive Ductal and Lobular Breast Carcinoma | 3 / 61 | 3.92E-7 | 5.599 | 3.117 | 380 (in top 2%) | No Associated Paper 2011/09/02 |
| TCGA Breast | Mixed Lobular and Ductal Breast Carcinoma | 7 / 61 | 1.26E-5 | 4.710 | 2.939 | 397 (in top 2%) | No Associated Paper 2011/09/02 |
